# Apropos of the Figures 1881

**Published:** 1881-02

**Authors:** 


Apropos of the figures 1881, the following is curious: In the Cabala nine is a mystic number, signifying "completion," and 1881 has the singular property of being divided thus—1 and 8 make 9, 8 and one make nine. In all the multiples of nine the total is nine. Thus: 9 times 2, 18, and one and eight make nine. Nine times three, twenty-seven and the figures 7 and 2 added make nine. Nine times four are 36, and 6 and three are 9, and so on indefinitely; and this is the only number which can be added into itself.

